# Community-acquired adult *Escherichia coli* meningitis leading to diagnosis of unrecognized retropharyngeal abscess and cervical spondylodiscitis: a case report

**DOI:** 10.1186/s12879-015-1310-4

**Published:** 2015-12-12

**Authors:** Rebekka Kohlmann, Andrey Nefedev, Martin Kaase, Sören G. Gatermann

**Affiliations:** Department of Medical Microbiology, Ruhr-University Bochum, Universitaetsstrasse 150, 44801 Bochum, Germany; Institute of Medical Laboratory Diagnostics (IML) Bochum GmbH, Castroper Strasse 45, 44791 Bochum, Germany; Department of Neurology, Evangelisches Krankenhaus Castrop-Rauxel, Grutholzallee 21, 44577 Castrop-Rauxel, Germany

**Keywords:** community-acquired adult meningitis, *Escherichia coli*, Gram-negative bacillary meningitis, retropharyngeal abscess, cervical spondylodiscitis, CTX-M-9 group ESBL, ST73

## Abstract

**Background:**

*Escherichia coli* is a rare cause of community-acquired meningitis in adults unless predisposing factors are present (e.g., previous penetrating cranio-cerebral injury or neurosurgery, immunosuppression, chronic alcoholism, history of cancer, diabetes mellitus, advanced age).

**Case presentation:**

We describe the case of a 53-year-old woman, resident in Germany, suffering from community-acquired bacterial meningitis caused by CTX-M-9 type extended spectrum β-lactamase producing *Escherichia coli*. Because typical predisposing factors were not apparent, pathogen identification resulted in expanded diagnostics to exclude a distant or contiguous primary focus. By magnetic resonance tomography, a previously unrecognized large retropharyngeal abscess with cervical spondylodiscitis was detected. In retrospect, the patient had complained about neck pain for a few weeks prior to meningitis onset, but the symptoms were interpreted as being related to a herniated disk. Meningitis and osteomyelitis resolved completely under surgical treatment and meropenem therapy.

**Conclusion:**

In case of adult *Escherichia coli* meningitis, underlying diseases should always be carefully excluded, especially if predisposing factors are not apparent.

## Background

Bacterial meningitis is a potentially life-threatening neurological emergency requiring early recognition of meningitis symptoms, rapid diagnostic evaluation of etiology, and immediate antimicrobial therapy. Its estimated annual incidence is 2–5 per 100,000 people in the Western world, and it ranks among the top ten causes of infection-related deaths worldwide and leads to permanent neurological sequelae in 30–50 % of the survivors [1]. In immunocompetent children and adults, meningitis is most often caused by *Streptococcus pneumoniae* and *Neisseria meningitidis* comprising over 80 % of meningitis cases, followed by *Streptococcus agalactiae*, *Haemophilus influenzae* and *Listeria monocytogenes* [2, 3]. In contrast, *Escherichia coli* is a rare cause of community-acquired adult meningitis comprising about 1 % of meningitis cases [3–5] even though it is a common pathogen in neonatal meningitis [6] and in the nosocomial setting, especially after penetrating cranio-cerebral injury or subsequent to neurosurgical procedures [7]. Community-acquired cases, however, occur almost exclusively in case of a preexisting risk factor, such as advanced age, history of cancer, immunosuppressive therapy, human immunodeficiency virus (HIV) infection, chronic alcoholism, or diabetes mellitus, and typically secondary to a distant or contiguous focus of infection, such as urinary tract infection, gastrointestinal infection, or otitis media [4, 5, 8–11]. Furthermore, the mortality rate is higher among patients with *Escherichia coli* meningitis than among those with other bacterial meningitides [4, 8].

We describe a community-acquired *Escherichia coli* adult meningitis case caused by a CTX-M-9 type extended spectrum β-lactamase (ESBL) producing strain. This case is of special interest because typical predisposing factors for *Escherichia coli* meningitis were not apparent. Thus, as a result of the unexpected pathogen identification, expanded diagnostics was performed to exclude a primary focus which led to diagnosis of previously unrecognized retropharyngeal abscess and cervical spondylodiscitis.

## Case presentation

A 53-year-old woman, resident in Germany, was admitted to the emergency department because of confusion, aphasia and agitation. Her partner reported that she had complained about neck pain for a few weeks which had been interpreted as being related to a herniated cervical disk in an X-ray examination seven days ago and treated with analgesics. Furthermore, the patient had suffered from unspecific flu-like symptoms in the previous week and intermittent diarrhea during the previous two months. Her past medical history revealed a chronic obstructive pulmonary disease based on nicotine addiction, a chronic pain syndrome following repeated vertebral disk herniation, obesity, and appendectomy as well as surgical repair of an epigastric and umbilical hernia six years ago. Because of the chronic lumbar pain, she had been under long-term morphine treatment with suspicion of morphine addiction, but had not received intraspinal steroid/analgesics injections for several months.

On physical examination, she had a body temperature of 38.6 °C; pulse rate and blood pressure were within the normal range. In the examination of respiratory system, cardiovascular system, and abdomen, no abnormalities were detected. The neurological examination also showed no abnormalities, especially no meningism and no neurological deficits, apart from the confusion, aphasia and agitation. Laboratory analysis at admission (Table 1) revealed a pronounced leukocytosis with an increase mainly in the neutrophil granulocytes, and a strongly elevated serum C-reactive protein level, indicating an infectious cause of the patient’s symptoms. Furthermore, a rise of lactate dehydrogenase, liver enzymes and cholestasis parameters, and a hyponatremia were observed. In the emergency department, a magnetic resonance tomography of the brain was performed, but demonstrated no abnormalities apart from first signs of a subcortical arteriosclerotic encephalopathy.

**Table 1:** Selected results of laboratory testing at admission and of lumbar puncture, respectively

Parameter (unit)	Normal range	Patient’s result
Blood leucocytes (10^3/μl)	4.0–9.0	33.5
Blood neutrophil granulocytes (%)	50–70	90, with a left shift
Blood lymphocytes (%)	25–40	3
Blood monocytes (%)	2–14	5
Blood erythrocytes (10^6/μl)	4.0–5.5	5.1
Blood thrombocytes (10^3/μl)	150–400	292
Serum C-reactive protein (mg/l)	0.0–5.0	163.9
Serum procalcitonin (ng/ml)	0.05–0.24	0.29
Serum creatine kinase (U/l)	0–190	63
Serum lactate dehydrogenase (U/l)	0–249	423
Serum alanine transaminase (U/l)	12–78	156
Serum aspartate transaminase (U/l)	0–35	38
Serum gamma-glutamyltransferase (U/l)	0–55	160
Serum total bilirubin (mg/dl)	0.0–1.0	1.84
Serum lipase (U/l)	73–393	303
Serum total protein (g/dl)	6.6–8.7	7.0
Blood glucose (mg/dl)	60–120	95
Serum creatinine (mg/dl)	0.5–1.1	0.7
Serum sodium (mval/l)	136–145	129
Serum potassium (mval/l)	3.5–5.1	4.3
Liquor leucocytes (/μl)	0–5	1,657
Liquor neutrophil granulocytes (%)	0–4	53.1
Liquor lymphocytes (%)	48–72	3.9
Liquor monocytes (%)	15–45	41.8
Liquor protein (mg/dl)	15–35	204.5
Liquor glucose (mg/dl)	25–60	0
Liquor lactate (mmol/l)	0.6–2.2	13.2

An immediate lumbar puncture was not successful because of the patient’s agitation, but was conducted on the second day of admission, when nuchal rigidity emerged. Because of strongly suspected bacterial meningitis, antimicrobial treatment with ceftriaxone (4 g daily in two divided doses) and ampicillin (12 g daily in six divided doses) was started. Analysis of the cerebrospinal fluid (Table 1) was consistent with bacterial meningitis, showing 1,657 leukocytes per μl, increased protein, undetectable glucose and increased lactate. Cerebrospinal fluid was also subjected to microscopic Gram stain examination and routine culture: The Gram stain revealed a high number of Gram-negative rods, and *Escherichia coli* grew on the culture plates. Species identification was performed by Matrix-Assisted Laser Desorption / Ionization (MALDI) Time of Flight (TOF) Mass Spectrometry (Bruker Daltonics, Bremen, Germany), and antimicrobial susceptibility testing was done by microdilution in accordance with European Committee on Antimicrobial Susceptibility Testing (EUCAST) recommendations [12], using pre-configured microtiter plates (MERLIN Diagnostika GmbH, Bornheim-Hersel, Germany). The *Escherichia coli* strain exhibited elevated minimum inhibitory concentrations (MICs) for aminopenicillins (amoxicillin: >128 mg/l, amoxicillin/clavulanic acid: 128/2 mg/l), oxyimino-cephalosporins (cefotaxime: 16 mg/l, ceftazidime: 2 mg/l), fourth-generation cephalosporins (cefepime: 64 mg/l) and monobactams (aztreonam: 16 mg/l), a synergy phenomenon in the double-disk synergy test with amoxicillin/clavulanic acid consistent with an ESBL, and a positive polymerase chain reaction (PCR) for *bla*_CTX-M-9 group_. In contrast, it showed low MICs to piperacillin/tazobactam (≤1/4 mg/l), carbapenems (imipenem: 0.0625 mg/l, meropenem: ≤0.03125 mg/l, ertapenem: ≤0.0625 mg/l, doripenem: ≤0.03125 mg/l) and fluoroquinolones (ciprofloxacin: ≤0.015625 mg/l, levofloxacin: ≤0.0625 mg/l), and was also susceptible to several other types of antibiotics such as aminoglycosides, trimethoprim/sulfamethoxazole, tigecycline, colistin and fosfomycin. In multilocus sequence typing (MLST), the strain was assigned to ST73. On the second day after admission, the initially taken blood cultures also flagged positive for ESBL-producing *Escherichia coli*. Of note, the patient’s ESBL carriage was not previously known. The antimicrobial therapy was switched to meropenem (6 g daily in three divided doses).

**Fig. 1:**
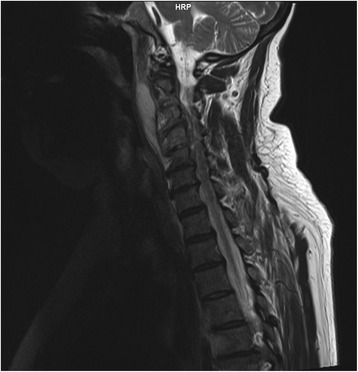
Result of magnetic resonance tomography of neck and spine. Magnetic resonance tomography, done on the seventh day after admission, showed the descending retropharyngeal abscess, measuring 7 cm × 3.5 cm × 1.5 cm, and the cervical spondylodiscitis at C2-C3 down to C4-C5

After pathogen identification, additional diagnostics was performed to exclude a primary focus of infection: Although a urine culture yielded an *Escherichia coli* strain having the same antimicrobial susceptibility pattern, the patient’s past medical history was negative for urinary tract infection symptoms. Furthermore, chest X-ray, abdominal ultrasound and transesophageal echocardiography conducted at admission and / or during the further hospital stay merely showed degenerative changes in the spine and an early-stage atherosclerosis, but did not reveal a primary focus. Thus, a magnetic resonance tomography of the spine was added in consideration of the patient’s neck pain even though that pain had already been attributed to a herniated cervical disk. However, magnetic resonance tomography showed a large descending retropharyngeal abscess with a cervical spondylodiscitis at C2-C3 down to C4-C5 and a secondary spinal canal stenosis (Fig. 1). Additionally, multiple disk protrusions were found, especially in the lumbar spine. Thus, the search for a source of meningitis, initiated because of the unexpected pathogen identification, led to diagnosis of previously unrecognized retropharyngeal abscess and cervical spondylodiscitis. In addition to continued meropenem treatment, the patient was immediately subjected to neurosurgery: The abscess was evacuated including polymethylmethacrylate-assisted ventral diskectomy. Intraoperative swabs also yielded the ESBL-producing *Escherichia coli*.

Initially, therapy was complicated by morphine withdrawal delirium requiring sedation of the patient. Then, the patient’s condition rapidly improved, and she had fully recovered without any neurological sequelae when meropenem treatment was stopped after 21 days (14 days after source-control surgery). Control computer tomography and magnetic resonance tomography three weeks and two months after admission, respectively, both proved complete remission of the retropharyngeal abscess and the spondylodiscitis. Nevertheless, hospitalization was prolonged because of secondary complications including prolonged dysphagia subsequent to the retropharyngeal abscess and several hospital-acquired infections. Specifically, the patient required mechanical ventilation in the course of two hospital-acquired pneumonia episodes, the first episode empirically treated with 7 days ceftriaxone and erythromycin, the second with 7 days imipenem. Then, she received 7 days vancomycin for treatment of *Enterococcus faecium* bacteremia. In addition, she developed bilateral trochanteric decubitus ulcers due to being bedridden for weeks. ESBL-producing *Escherichia coli* was isolated from those decubitus which were subsequently treated surgically and with 10 days ciprofloxacin. Finally, the patient received 12 days caspofungin because of candidemia. Of the antimicrobials used for therapy of the hospital-acquired infections, imipenem and ciprofloxacin also had in vitro activity against the meningitis-causing *Escherichia coli* strain. However, neither clinical nor radiological signs of a meningitis relapse occurred after cessation of meropenem, which may indicate that the meningitis had already been cured at the time-point of meropenem cessation. After all, the patient was discharged from hospital in good health three months after admission.

## Discussion

Previous case series on adult Gram-negative bacillary meningitis usually include or even focus on nosocomial meningitis cases which occur particularly subsequent to penetrating cranio-cerebral injury or neurosurgery [4, 5, 8–11, 13, 14], whereas *Escherichia coli* is a rare cause of community-acquired adult meningitis [3–5]. However, in the present case there was no evidence of a nosocomial meningitis origin. In particular, the patient had never received any intraspinal injections close to the cervical spine and an infiltration of the lumbar spine dated back several months. Considering only the community-acquired adult Gram-negative bacillary meningitis cases included in the above mentioned case series, it is striking that most cases, namely about 75 % [4, 8] to 95 % [5, 9], occurred in patients with predisposing risk factors such as advanced age, history of cancer, immunosuppressive therapy, HIV infection, chronic alcoholism, or diabetes mellitus. However, our patient did not display any of those risk factors. Moreover, about 75 % of the cases [4, 8] occurred secondary to a distant or contiguous infection (e.g., urinary tract infection, gastrointestinal infection, or otitis media). Thus, in the present case, diagnostics was extended after pathogen identification to exclude a primary focus of infection, which led to diagnosis of the previously unrecognized retropharyngeal abscess with cervical spondylodiscitis. Consequently, our case report highlights the importance of searching for a source of meningitis in cases of *Escherichia coli* adult meningitis, especially if typical risk factors are not apparent.

Probably, the patient’s meningitis as well as the cervical spondylodiscitis had arisen through contiguous spread from the retropharyngeal abscess. However, retropharyngeal abscesses are mainly polymicrobial infections containing common oropharyngeal microflora but not Enterobacteriaceae. Typically, they are caused by anaerobic organisms and beta-hemolytic streptococci (especially *Streptococcus pyogenes*), followed by viridans group streptococci, *Staphylococcus aureus*, *Haemophilus influenzae* and *Streptococcus pneumoniae* [15, 16]. Furthermore, the majority of spondylodiscitis cases are acquired by hematogenous spread, followed by direct external inoculation, whereas spread from contiguous tissues is comparably rare [17]. In hematogenous spondylodiscitis, *Staphylococcus aureus* is most commonly identified (apart from tuberculosis which is the leading cause worldwide), but multiple other pathogens may also be causative agents. In case of direct external inoculation, cutaneous organisms may also be found. Thus, even though *Escherichia coli* may be no rarity in hematogenous spondylodiscitis, in cases acquired through contiguous spread based on a retropharyngeal abscess one would expect pathogens from the oropharyngeal microflora rather than *Escherichia coli*.

Because *Escherichia coli* is no typical cause of a retropharyngeal abscess, one may suppose a previous unrecognized *Escherichia coli* bacteremia. However, a precedent distant focus of infection was not found in the present case and, therefore, the reason for the retropharyngeal abscess remains elusive. In particular, the patient’s past medical history was negative for urinary tract infection symptoms even though one urine culture yielded the ESBL-producing *Escherichia coli* strain. The latter may be explained by colonization of the urinary tract subsequent to an assumable colonization of the gastrointestinal tract, or, less likely, may result from the bacteremia present during the meningitis episode. The hypothesis of lasting gastrointestinal and urinary tract colonization is supported by the fact that the ESBL-producing *Escherichia coli* strain was detected from the patient’s bilateral trochanteric decubitus ulcers which occurred only after full meningitis remission and were, thus, probably not related to the meningitis episode. In addition, normal findings in abdominal ultrasound argue against a precedent gastrointestinal infection although the patient reported that she had had intermittent diarrhea during the previous two months. The abnormal liver function test results may, in consideration of previously normal values, also be interpreted as accompanying sepsis-related changes because hepatic dysfunction represents a common manifestation during sepsis process [18]. Furthermore, prior abdominal surgery as a potential source of *Escherichia coli* bacteremia dated back several years.

Of note, the presented community-acquired *Escherichia coli* adult meningitis was caused by an ESBL-producing strain. This may increase the significance of the present case report because ESBL-producing strains have been rarely identified in *Escherichia coli* meningitis, in community-acquired cases even less than in the nosocomial setting [13, 19, 20]. Our *Escherichia coli* isolate produced a CTX-M-9 group ESBL and was assigned to ST73 in MLST. To the best of our knowledge, this is the first report of a CTX-M-9 group ESBL in the setting of *Escherichia coli* meningitis. CTX-M-9 group ESBLs are comparably infrequent in Germany, accounting for only 7–12 % of ESBLs detected in *Escherichia coli* isolates (for comparison: CTX-M-1-group 80–93 %) [21, 22]. In contrast, ST73, along with ST131, ST69, and ST95, belongs to the major pandemic clonal lineages as was summarized in a recent review [23]. Interestingly, in some of the reviewed studies providing appropriate subanalyses, ST73 strains tended to occur more frequently in community-acquired than in hospital-acquired infections [24, 25], but were only rarely (0–2 %) associated with ESBL production [24, 26].

## Conclusion

Community-acquired *Escherichia coli* adult meningitis is a rare entity which usually occurs merely in case of predisposing factors. If typical risk factors are not apparent, it may be prudent to extend diagnostics for exclusion of underlying diseases and precedent infections.

### Consent to publish

Informed consent was obtained from the patient for publication of this case report and any accompanying images.
